# Assessment of cortical reorganization and preserved function in phantom limb pain: a methodological perspective

**DOI:** 10.1038/s41598-020-68206-9

**Published:** 2020-07-13

**Authors:** Jamila Andoh, Christopher Milde, Martin Diers, Robin Bekrater-Bodmann, Jörg Trojan, Xaver Fuchs, Susanne Becker, Simon Desch, Herta Flor

**Affiliations:** 1 Department of Cognitive and Clinical Neuroscience, Central Institute of Mental Health, Medical Faculty Mannheim, Heidelberg University, J5, 68159 Mannheim, Germany; 2 Department of Psychology, University of Koblenz-Landau, Landau, Germany; 3 Department of Psychosomatic Medicine and Psychotherapy, LWL University Hospital, Ruhr-University Bochum, Bochum, Germany; 4 Biopsychology and Cognitive Neuroscience, Faculty of Psychology and Sports Science, Bielefeld University, Bielefeld, Germany

**Keywords:** Neuroscience, Psychology

## Abstract

Phantom limb pain (PLP) has been associated with reorganization in primary somatosensory cortex (S1) and preserved S1 function. Here we examined if methodological differences in the assessment of cortical representations might explain these findings. We used functional magnetic resonance imaging during a virtual reality movement task, analogous to the classical mirror box task, in twenty amputees with and without PLP and twenty matched healthy controls. We assessed the relationship between task-related activation maxima and PLP intensity in S1 and motor cortex (M1) in individually-defined or group-conjoint regions of interest (ROI) (overlap of task-related activation between the groups). We also measured cortical distances between both locations and correlated them with PLP intensity. Amputees compared to controls showed significantly increased activation in M1, S1 and S1M1 unrelated to PLP. Neural activity in M1 was positively related to PLP intensity in amputees with PLP when a group-conjoint ROI was chosen. The location of activation maxima differed between groups in S1 and M1. Cortical distance measures were unrelated to PLP. These findings suggest that sensory and motor maps differentially relate to PLP and that methodological differences might explain discrepant findings in the literature.

## Introduction

Extensive research has shown that increased use or sensory stimulation of limbs results in an enlarged cortical representation in contralateral primary somatosensory (S1) and motor (M1) cortices^1,2^. In contrast, reduced limb use or injury such as deafferentation leads to a decrease of input to the sensorimotor cortices and a reduced cortical representation, and often an expansion of the representation of adjacent body parts^3,4^. Such shifts in boundaries of the body map, termed cortical reorganisation, have been shown following arm deafferentation, indicating that the hand area became responsive to inputs from the face whose representation neighbors the hand area^5^. Recent studies revealed, however, that the extent of intracortical projections across the hand–face boundary in dorsal column lesions in monkeys was small^6^, suggesting that reorganization might involve much broader changes than those in S1.

In humans, alterations in primary sensorimotor areas have been associated with perceptual and behavioral changes^7–9^. In particular, the amount of reorganization in S1 after limb amputation has been found to be positively related to phantom limb pain (PLP)^10,11^ although this finding has not been consistently replicated^12^.

Using a motor task involving movements of the phantom hand (or motor imagery for amputees who could not move the phantom and matched two-handers) a positive relationship between the magnitude of PLP and the peak intensity of brain activation in the sensorimotor region representing the phantom hand was reported^13^. In contrast to studies reporting maladaptive plasticity, this study suggested preserved representation of the phantom limb in S1 as a correlate of PLP. The meaning of these two seemingly contradictory findings is the basis of an ongoing scientific debate^12,14–19^.

Maladaptive plasticity was first shown by examining neural activity related to passive sensory stimulation applied to the mouth and the intact hand using magnetoencephalography^10,11^. Flor et al.^10^ applied tactile stimuli to the lips and the fingers of unilateral upper limb amputees and found a displacement of the lip representation towards the hand representation in S1, which was related to the magnitude of PLP, such that the closer the lip was to the hand somatosensory area, the more intense the PLP. This finding was also replicated using tasks in the motor domain implementing either executed or imagined phantom hand movements. An expansion of neural activity from the lip into the hand areas of both S1 and M1 was found only in amputees with PLP, and this shift was positively correlated with PLP intensity^18,20,21^. Diers et al. showed that mirrored movements of the intact hand failed to activate the cortical representation of the phantom limb in S1 and M1 in unilateral upper limb amputees with PLP, whereas the phantom limb was activated in amputees without PLP^22,23^. The more activation in the phantom cortex, the less intense PLP was observed. These findings seem in opposition to those reported by Makin et al.^13^, who found a positive association between PLP and activation in the sensorimotor (S1M1) phantom cortex.

In Makin et al.^13^, the cortical representation of the phantom hand was defined using a conjunction analysis to reveal shared neural activity between execution or imagery of phantom movements in brain areas encompassing precentral and postcentral gyri (i.e., M1 and S1) in 18 participants with upper limb amputation (16 with, 2 without PLP), 11 persons with a congenital upper limb deficiency not reporting PLP and in 22 two-handed controls^13^. Thus, the neural activity the authors correlated with PLP was based on joint activation maxima of all groups irrespective of whether there had been reorganization in the hand representations in M1 and S1 in the PLP group^3,20,24^. In this case, the use of a conjunction analysis might not have captured the current (post-amputation) hand representation but the original pre-amputation location.

An additional important factor when examining the cortical representation of the phantom hand relates to the task being used. Various methods have been implemented in the literature using either imagery-, movement-, mirrored-, hypnosis-elicited phantoms or evoked-phantoms sensations^25–27^ or their combination^13,28^.

The localization of the motor/somatosensory representation of the phantom hand remains, however, challenging, which is not only related to the fact that the hand to be mapped is absent, but also to the heterogeneity of phantom sensations among amputees. Some amputees do not experience vivid phantom sensations and might not be able to perform phantom movements^13^. In such cases, studies combined neural activations resulting from different tasks, such as execution or imagery of movements of the phantom hand^13,28^.

Noteworthy, execution of phantom movements is often accompanied by activity in the muscles of the residual limb^29^, therefore activity in the motor cortex might result from associated residual limb muscle movements, especially if the participants are trained to use these muscles during phantom movements^13^. The role of the residual limb during the execution of phantom hand movements has also been shown using transcranial magnetic stimulation applied over the representation of the amputated hand^30^. This evoked not only phantom movements but also contractions in the muscles of the residual limb, raising the question of whether the phantom movements involuntarily involved the proximal arm muscles, potentially modulating activity in S1M1^30^.

Other studies used tasks based on imagery of phantom movements^20,21^, or mirrored movement task^12^, in which the intact hand was moved in front of a mirror, creating the illusion of a moving phantom limb in amputees.

However, both processes, motor imagery and motor execution, have been shown to have different neural representations^26,31^ and might therefore show a different relationship with PLP.

An additional factor to take into account relates to brain areas being investigated, for example, S1 or M1 and their definition based on structural or functional images, anatomical atlas, or histological investigations.

For instance, S1 and M1 have been shown to be differently activated by movements and imagery of movements of the phantom hand, with S1 being activated by both movements and imagination of movements of the phantom hand, and M1 being activated by movements and not by imagery^26^. These findings highlight differences in neural activity between amputees when combining execution and imagery of phantom movements and could explain potential differences in the results reported in the literature^13,26^.

Moreover, the definition of the ROI might vary depending on the brain parcellation method used and could also explain some inconsistencies across studies. For example, most studies defined S1 and M1 based on anatomical atlases (e.g. automated anatomical labelling^32^, MINC^33^, Harvard–Oxford^25^). Other studies defined regions of interest (ROIs) for S1 and M1 based on overlap of functional activity (*conjunction*) between groups of controls and amputees^13,15^. Although some efforts have been made to consolidate these atlases^34^, more work is needed to understand similarities and differences between studies.

Since a number of methodological factors might influence the activity in sensory and motor representations in amputees and thus the relation of neural activity to PLP, in the present study, we used fMRI combined with a standardized virtual reality movement task^23^, which allowed for identical implementation in upper limb amputees (virtual movement of the phantom) as well as two-handed controls (where movement of the contralateral hand was shown). Further, we trained the amputees not to move their residual limb before participating in the phantom movement task, which was verified by electromyographic assessments on the residual limb in a subsample of amputees.

We examined task-related neural activity separately in the primary somatosensory (S1), primary motor (M1), and primary sensorimotor cortices (S1M1) as previously demonstrated^13^ in the hemisphere contralateral to the amputation (or side-matched hemisphere in non-amputated controls). We also assessed differences in neural activity between amputees with and without PLP and healthy controls, and their relationship to PLP intensity using cortical distance measures and we employed both group-conjoint and individual specific regions of interest (ROI). Our hypotheses stated that the neural activity resulting from the virtual reality movement task differentially involves S1 and M1 in amputees with and without PLP, and in controls. In addition, we expected to find different relationships between neural activity and PLP depending on the approach used, i.e. using activation maxima (either individually defined or based on conjoint activation), or if one examines M1 or S1 cortices, or calculating distance measures.

## Methods

### Participants

Twenty unilateral upper limb amputees (age (M ± SD*)* = 51.40 ± 12.54 years; 10 amputees with PLP (PLP group), including four right-arm amputees, and 10 amputees without PLP (nonPLP group), including six right-arm amputees, were recruited.

Time since amputation did not differ significantly between PLP and nonPLP (t(17) = 0.81, *p* = 0.43 (time since amputation (M ± SD) = 23.6 ± 15.49 years for nonPLP; (M ± SD) = 18.60 ± 8.55 years for PLP). Fifteen amputees reported phantom limb awareness (n = 10 in the PLP group). In addition, 20 non-amputated controls without chronic pain were recruited (age (M ± SD*)* = 51.55 ± 12.62 years). Non-amputated controls were matched to the amputees in terms of age, handedness and sex (see Table 1).

**Table 1:** Characteristics of amputees and controls: MPI: Pain Intensity Scale modified to assess phantom pain based on the West Haven-Yale Multidimensional Pain Inventory^10,39^.

	PLP group	nonPLP group	Controls
Age (M, range)	51.5, [69–26]	51.3, [67–20]	51.5, 21–70
Sex (N females)	3	2	5
Age at amputation (M ± SD)	32.90 ± 13.04	21.5 ± 12.48	N/A
Side of amputation (L/R)	6/4	4/6	N/A
MPI Pain Intensity Scale (M ± SD)	2.70 ± 0.71	0	N/A
PLP before fMRI	2.95 ± 2.32	0	N/A
PLP after fMRI	2.35 ± 2.00	0	N/A
Item1 (M ± SD)	4.86 ± 2.79	3.00 ± 2.65	3.04 ± 2.45
Item2 (M ± SD)	3.86 ± 2.23	2.29 ± 1.93	2.29 ± 1.50
Item3 (M ± SD)	6.57 ± 2.79	4.86 ± 2.54	5.04 ± 3.26
Analgesic medication (N)	0	3	N/A
Other medication (N)	3	4	N/A

Analgesic medication (Tramadol hydrochloride). Other medication (hypertension: Ramipril, Micardis, Metohexal, Beloc Zok, Isoptin, Kerlone, betaBlo). PLP group: amputees with phantom limb pain, nonPLP group: amputees without phantom limb pain. L/R: left / right-arm amputees*.*

Items 1–3 refer to ratings performed by the participants at the end of the MRI evaluate the perception of the moving hand as their own.

Item1: ratings of how much participants felt that the movement they saw was related to the phantom/matched hand; Item2: ratings of how vivid the movement participants saw was perceived in their amputated hand; Item3: how much they had a feeling to directly view the phantom hand (each item was rated using a numerical rating scale ranging from 0 = no sensation to 10 = very strong sensation).

There was no significant age difference between the control and the amputee group (t(38) = − 0.04, *p* = 0.97). There was also no significant age difference between the PLP and the nonPLP group (mean age ± SD PLP group 51.30 ± 12.09; nonPLP group 51.50 ± 13.64, t(18) = − 0.03, *p* = 0.97).

Ethical committee approval was received from the Institutional Review Board of the Medical Faculty Mannheim, Heidelberg University, and written informed consent was obtained from all participants. The study protocol adhered to the Declaration of Helsinki.

### Psychometric assessment of phantom phenomena

The amputees participated in a psychometric evaluation, during which they were tested for the presence of mental disorders using a screening version of the Structured Clinical Interview for the Diagnostic and Statistical Manual of Mental Disorders (DSM-IV)^35^. A quantitative measure of psychological distress was obtained by the German version of the Brief Symptom Checklist^36^. We further assessed anxiety and depression by the Hospital Anxiety and Depression Scale^37^, and catastrophizing was examined by the Pain-Related Self-Statements Scale^38^. In addition, we also carried out a structured interview about amputation characteristics and phantom phenomena^39^, during which we specifically targeted duration, intensity, and frequency of painful and nonpainful phantom phenomena as well as painful and nonpainful residual limb phenomena^39^. In addition, the German version of the West Haven–Yale Multidimensional Pain Inventory (MPI;^40,41^) was used in a modified version that separately assessed PLP and residual limb pain^10^. PLP intensity was defined by the MPI pain intensity subscale.

### Experimental procedure

We developed a virtual reality movement task to be carried out in an MR scanner^23^. The task consisted of a virtual reality environment in which participants were seeing a virtual body from a first-person perspective and the scanner bore through MR-compatible goggles (VisuaStimDigital, Resonance Technology, Inc., Northridge, CA, USA). We used the 3D-graphical simulation program KISMET (Kinematic Simulation, Monitoring and off-line programming Environment for Telerobotics), developed at the KIT (Karlsruhe Institute of Technology)^23,42,43^. The hardware and the full virtual reality environment (MR-glove, acquisition hard- and software, simulation software (KISMET) and models) were custom-built by the KIT^23,44^.

The participants wore a glove on their intact hand (matched side for non-amputated controls) capable of tracing movements that were transformed into synchronous movements of an avatar hand. We also had a mirror condition, during which the avatar hand was mirrored, and we presented both the right and left hands of the avatar in the virtual environment (analogous to the classical mirror box)^23^. Participants were instructed to perform open-close movements with the intact/matched hand at a frequency of 0.5 Hz, paced by tones presented via earphones. Participants were asked to observe the movement of the virtual hand carefully and were instructed to perceive this movement as movement of their phantom/matched hand^23^. This set-up created a uniform perception of movement of the phantom hand/intact hand with identical visual input in the amputees and controls and ensured task consistency between amputees and two-handed controls. To evaluate to what extent the participants felt that the moving hand was perceived as their own, we asked them at the end of the MR scan to rate three items asking (a) how much they felt that the movement they saw was related to the phantom (b) how vivid the movement they saw was perceived in their amputated hand (c) and how much they had a feeling to directly view the phantom hand (each item was rated using a numerical rating scale ranging from 0 = no sensation to 10 = very strong sensation). The two-handed controls were asked equivalent questions about the hand they saw on the contralateral side.

The task consisted of alternating 19.8 s periods of movement and rest, which were repeated six times.

The participants were shown if they moved the residual limb along with the intact arm in a training phase and were discouraged from moving the muscles of the residual limb along with the paced open-close hand movements. Electromyographic activity of the residual limb was also recorded during the MRI session (Brain Products GmbH, Munich, Germany) with a 5000 Hz sampling frequency in a subsample of n = 12 amputees. EMG data could not be collected on all amputees because, in some subjects that data could not be used (e.g. depending on their level of amputation, number of muscles remaining in the stump, or artefacts resulting from the simultaneous recordings) and for some amputees this was too demanding.

### MRI acquisition and preprocessing

MRI data were acquired using a Siemens 3 T TRIO scanner (Siemens AG, Erlangen, Germany) in combination with a 12-channel radiofrequency head-coil. During the virtual reality movement task, 80 echo planar imaging (EPI) volumes were acquired for each participant (2.3 × 2.3 × 2.3 mm, TR/TE = 3,300/45 ms, FOV = 220 mm, matrix size = 220 × 220), comprising 40 slices covering the whole brain. A high-resolution 3D magnetization prepared rapid gradient echo image (MPRAGE, 1 mm isotropic voxel, TR/TE = 2,300/2.98 ms) was acquired for anatomical reference.

### Data analysis

#### Analysis of individual fMRI data and definition of individual regions of interest (ROIind)

Functional imaging data acquired during the mirror condition were analyzed using the FMRIB Software Library (FSL 5.0.9)^45^. For all datasets, motion correction was applied using MCFLIRT^46^ and motion-correction parameters were used as nuisance regressors in the design matrix. Spatial smoothing was performed using a 5 mm isotropic Gaussian kernel of full-width at half-maximum (FWHM) and high-pass temporal filtering was applied using a Gaussian-weighted least square straight line fitting at 100 s cut-off. Registration was performed using a 2-step procedure: EPI images from each scan were first registered to the high resolution T1-weighted structural image where non-brain structures were removed using Brain Extraction Tool^47^. EPI images were then registered to the standard MNI152 template using 12-parameter affine transformations. The fMRI statistical analysis was carried out using FEAT (fMRI Expert Analysis Tool^45^). Data from each participant were analyzed separately at a first level analysis. Trials for the motor task were modeled as a single factor of interest and were convolved with a canonical Gaussian hemodynamic response function and were entered as a predictor into a general linear model. Data collected for right-sided (n = 10) amputees were mirror-reversed across the mid-sagittal plane prior to any analysis so that the deafferented hemisphere was consistently aligned (virtually, all amputees became left-sided amputees). Therefore, in all the following analyses we examined the right hemisphere corresponding to the missing left hand.

We defined an individual virtual phantom/matched hand movement ROI (ROIind) based on the location of the individual peak voxel, which was used as the center of a spherical ROI (5 mm radius), see Table 2. This approach is assumed to identify individual-related variability in the peak of activation and location of body site representations, which can be expected from previous results showing high variability in locations of peak activity in S1 and M1 in amputees with PLP compared to those without PLP and two-handed controls (e.g.,^10,18^).

**Table 2:** Coordinates of the peak voxel for each participant transformed in MNI 152 space for M1, S1 and S1M1 ROIs. PLP group: amputees with phantom pain; nonPLP group: amputees without phantom pain.

Groups	Group	ID	X(S1)	Y(S1)	Z(S1)	X(M1)	Y(M1)	Z(M1)	X(S1M1)	Y(S1M1)	Z(S1M1)
Amputees	nonPLP group	A01	30.8	− 36.6	54	39.9	− 18.4	61.9	39.9	− 18.4	61.9
		A02	45.8	− 26.2	59	42.7	− 22.9	68.6	40.9	− 26	55.3
		A03	44.4	− 17.1	55.7	49	− 15.8	62.2	49	− 15.8	62.2
		A04	41	− 17.4	55.4	31	− 27.5	56	31	− 27.5	56
		A05	42.3	− 17.6	53.9	46.8	− 12.9	53.6	49.4	− 15.1	60.1
		A06	36.9	− 28.3	50.8	11.5	− 24.5	60.6	36.8	− 24.7	75.5
		A07	60	− 12.4	27.4	37.9	− 19.4	59.0	37.9	− 19.4	59
		A08	39.6	− 33.5	59.7	22.3	− 29.9	73.4	19.8	− 32.2	77.5
		A09	42.9	− 21.8	51.8	51.3	− 2.3	39.7	51.9	− 13.8	48
		A10	39.2	− 32.3	58.3	52.4	1.8	24.3	39.3	− 34.7	61.9
	PLP group	A11	37	− 33.1	52.8	61.5	− 15.6	46.8	37.5	− 34.6	66.1
		A12	36.9	− 33.1	53.2	49.8	− 15.3	44.0	49.8	− 15.3	44
		A13	45.1	− 22.3	58.9	40.2	− 26.9	62.6	45.1	− 22.3	58.9
		A14	21	− 32.1	71	20.9	− 31.7	74.2	10.5	− 25.6	75.3
		A15	58.3	− 11.2	36.2	59.9	− 11.1	46.9	59.9	− 11.1	46.9
		A16	37.3	− 35.2	58.4	26.9	− 24.6	54.8	29.4	− 42.2	77
		A17	27.4	− 38.5	69.9	36.6	− 30.8	68.8	21.9	− 32.4	78.9
		A18	39.8	− 36	58	42.0	− 2.4	40.1	10.2	− 23.1	81
		A19	37.7	− 31.9	52.8	42.5	− 16.1	59.7	37.7	− 31.9	52.8
		A20	43.9	− 20.3	59.6	41.5	− 22.2	63.3	31.9	− 27.5	77.8
Controls		C01	40.6	− 17.1	46.7	64.6	− 6.4	33.4	64.6	− 6.4	33.4
		C02	54.5	− 4.4	37.3	54.5	0.6	36.5	54.5	− 1.9	36.9
		C03	44.3	− 24.5	53.9	52.0	− 7.2	45.9	52	− 7.2	45.9
		C04	42.6	− 13.5	51.4	53.6	0.9	35.9	54.2	− 3.7	39.7
		C05	52.7	− 16.2	37.9	52.0	− 5.5	43.5	51.9	− 7.4	47.3
		C06	42.5	− 31.5	60.4	54.5	− 16.7	46.2	42.5	− 31.5	60.4
		C07	55.3	− 17.8	41	36.9	− 14.6	53.4	6.7	− 24.2	75.6
		C08	61.2	− 15.1	30.3	54.6	0.8	36.9	12.2	− 31.9	85.1
		C09	53.4	− 18.4	34.1	50.6	− 13.6	37.0	53.4	− 18.4	34.1
		C10	62.8	1.6	15.8	54.5	− 1.9	36.9	42.9	− 8.5	50.1
		C11	32.6	− 32.4	54.4	52.0	− 7.2	45.9	40.2	− 10.8	51.7
		C12	58	− 15.3	28.1	54.2	− 3.7	39.7	60.2	− 8.9	47.5
		C13	33.2	− 33.9	48	51.9	− 7.4	47.3	33.2	− 33.9	48
		C14	35.5	− 36.8	62.9	49.5	1.9	29.7	23	− 40	78
		C15	37.1	− 35.9	57.8	58.7	− 13.0	43.5	37.1	− 35.9	57.8
		C16	44.4	− 12.5	54.5	53.4	− 18.4	34.1	49.3	− 14.7	61.2
		C17	51.5	− 8	39.8	49.4	− 5.3	42.9	47.2	− 6.7	49.9
		C18	64.3	3.3	15.2	50.3	− 18.3	34.2	64.3	3.3	15.2
		C19	25.8	− 32.8	77.5	53.8	− 6.2	41.4	25.8	− 32.8	77.5
		C20	65.1	0.9	16.8	62.8	0.7	16.9	53.8	− 6.2	41.4

#### Definition of the group-conjoint ROI (ROIconj)

We then defined a conjunction ROI (ROIconj), in accordance to Makin et al.^13^, namely the conjunction of neural activity associated with the virtual phantom/matched hand movement between PLP, nonPLP, and two-handed individuals. For this purpose, a second level analysis was carried out for each group using a mixed-effects analysis as implemented in FLAME (FMRIB's Local Analysis of Mixed Effects). Then ROIconj was obtained based on the conjunction of the three groups^48^ separately within the S1 and M1 using probabilistic maps provided by the Juelich histological atlas^49^. Additionally, to relate our findings to previous work^13^, we also examined ROIconj in S1M1, which was defined by the combination of the S1 and M1 maps.

Then, we extracted the percent BOLD signal change (%BSC) for each participant and for each ROI (ROIconj, ROIind) in S1, M1 and S1M1 using the FSL Featquery tool^45^. The %BSC for each ROI was compared between amputees and controls, and between PLP and nonPLP groups using one-way analysis of variance (ANOVA).

For both individual and group analyses, areas of significant fMRI responses were determined using clusters identified by a *z* > 2.3 threshold and a Threshold-Free Cluster Enhancement (TFCE)-FWE of *p* < 0.05^50,51^.

#### Correlations between fMRI activation and PLP intensity

Correlation analyses were performed using R package version 3.5.2 (https://www.r-project.org/). PLP ratings were inspected for normality violations using the Shapiro–Wilk test. PLP intensity was normally distributed in the PLP group (*p* = 0.98). Homogeneity of variances was tested using Levene’s test. If this assumption was violated, equal variance was not assumed and the data were reported accordingly. In the case of zero-clustered data (in nonPLP group), (Levene's Test *p* = 0.004), Pearson correlation have been shown to perform better than the Spearman rank correlations^52^. We also tested the correlations performed using bootstrap estimates and confidence intervals. Furthermore, we used parametric tests because they are known to be more robust than non-parametric tests when applied to small sample sizes or zero-clustered data^53^. *Post-hoc* t-tests were conducted when necessary and FDR-corrected p-values were used to correct for multiple comparisons. Statistical thresholding was done at *p* = 0.05.

Correlation analyses were carried out between PLP intensity and the %BSC extracted from ROIconj and ROIind for S1, M1 and S1M1 in the deafferented hemisphere in amputees and the corresponding hemisphere in controls.

We also tested if these correlation coefficients between PLP intensity and the %BSC extracted from S1, M1 and S1M1 were statistically different between ROIconj and ROIind (cocor, R package for dependent sample)^54^.

In addition, we recalculated ROIconj using the overlap of task-neural activity between amputees and a subsample of the controls (n = 12) with a location of the hand area > z = 40. Although the morphology of the region of the primary motor cortex in the human brain is variable (between hemispheres and between individuals^55^), previous work on the quantitative cytoarchitectonic analysis of BA4 and neuroimaging studies indicated that BA4 has a ventral-dorsal axis z > 40^55–58^. There was no significant age difference between the subsample of the controls and the amputees (mean age ± SD subsample of controls 51.40 ± 12.54; amputees 54.33 ± 9.91, t(28) = − 0.73, *p* = 0.47). There was also no significant age difference between the subsample of the controls and the nonPLP group (t(16) = 0.57), and no significant age difference between the subsample of the controls and the PLP group (t(17) = 0.56).

#### Correlations between fMRI activation and electromyography (EMG) signal

For the EMG, we carried out a correlation between electromyography activity from the residual limb and task-dependent neural activity during the virtual reality movement task.

#### Correlation between cortical distances and PLP intensity

Cortical distances were calculated using Euclidean distances between the ROIind and ROIconj separately for S1, M1, and S1M1 and for each group, and examined the relationship between Euclidean distances and PLP intensity. Similar analyses were carried out using cortical distances in the mediolateral direction.

#### Group comparisons for the perceptual data during the task

Finally, in order to assess the percept of the virtual phantom hand movement during the task between groups, we compared the ratings of the three items between the groups, and between PLP and nonPLP groups. The ratings were not normally distributed, therefore we used non-parametric alternatives (Kruskal–Wallis). For all analyses, effect sizes were reported.

## Results

### Overlap of the task-neural activity between the three groups

**Figure 1:**
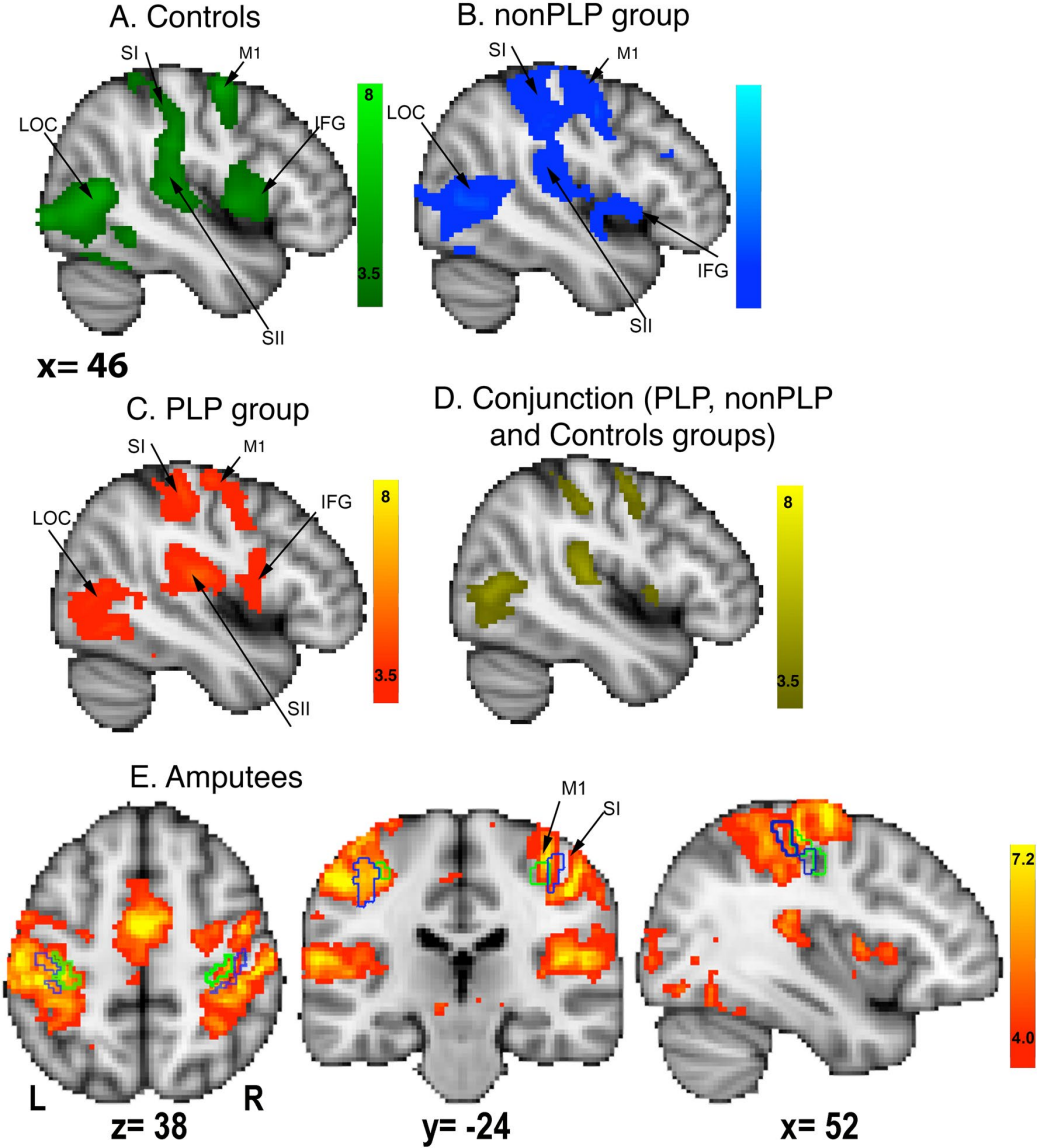
Mean task-related activity during the virtual phantom task in the hemisphere contralateral to amputation (matched in healthy controls) for **a** the control group (green-lightgreen), **b** the nonPLP group (blue-lightblue) and **c** the PLP group (red-yellow). **d** Conjunction of task-related neural activity between the controls, PLP, and nonPLP groups (yellow). **e** Mean neural activity during the virtual phantom task in the amputee group. The green contours indicate the borders of the primary motor cortex (BA4p) and the blue contours indicate the borders for the primary somatosensory cortex (BA3b) in both hemispheres based on Juelich histological atlas. Activations are mapped on a MNI152 template provided by FSL, with FWE *p* < 0.05, TFCE, z > 2.3 threshold. Abbreviations: SI: primary somatosensory cortex, M1: primary motor cortex, LOC: lateral occipital cortex, IFG: inferior frontal gyrus, SII: secondary somatosensory cortex.

Separate analyses for the PLP, nonPLP and two-handed controls showed neural activity in primary motor and somatosensory cortices, secondary somatosensory cortex, inferior frontal gyrus and lateral occipital cortices in the hemisphere contralateral to the amputation/matched hemisphere (Fig. 1a–c). Brain areas activated by the virtual movement task, which showed overlap between the three groups (i.e., that were commonly activated in the three groups), are shown in Fig. 1d. We found that the virtual phantom/hand movement task induced bilateral neural activity in primary motor and somatosensory cortices as defined by the Juelich histological atlas (https://fsl.fmrib.ox.ac.uk/fsl/fslwiki/Atlases/Juelich) in area BA3b and BA4p, respectively (Fig. 1e). Additional information is provided in Supplementary Fig. 1 for task-related neural activity in the hemisphere contralateral to the intact hand, and Supplementary Table 1 for mean values and standard deviations of %BSC in S1, M1 and S1M1 for the three groups. There was no significant group difference in task-related activity in the hemisphere ipsilateral to the amputation/matched hemisphere (see Supplementary Fig. 2).

### Comparison of neural activity between the three groups in ROIconj and ROIind

The %BSCs for the ROIconj in S1, M1 or S1M1 did not significantly differ between amputees (PLP and nonPLP groups) and controls (S1: F(1,38) = 1.84, *p* = 0.18; M1: F(1,38) = 3.15, *p* = 0.08; S1M1: F(1,38) = 2.87, *p* = 0.10). In addition, no significant differences in %BSC were found between PLP and nonPLP using ROIconj (S1: F(1,18) = 0.002, *p* = 0.97; M1: F(1,18) = 0.87, *p* = 0.36; S1M1: F(1,18) = 0.20, *p* = 0.66). Using ROIind, we found that amputees had a significantly increased %BSC in S1, M1 and S1M1 compared to controls (S1: F(1,38) = 15.19, *p* < 0.001, partial eta square η_p_^2^ = 0.29); M1: F(1,38) = 9.98, *p* = 0.003, η_p_^2^ = 0.21; S1M1: F(1,8) = 13.44, *p* < 0.001, η_p_^2^ = 0.26). However, no significant differences in %BSC were found between the PLP and the nonPLP groups using ROIind (S1: F(1,18) = 0.090 *p* = 0.77; M1: F(1,18) = 0.74, *p* = 0.40; S1M1: F(1,18) = 0.48, *p* = 0.50). See Supplementary Table 1 for mean values and standard deviations.

### Relationship between PLP and neural activity in ROIconj and ROIind

**Figure 2:**
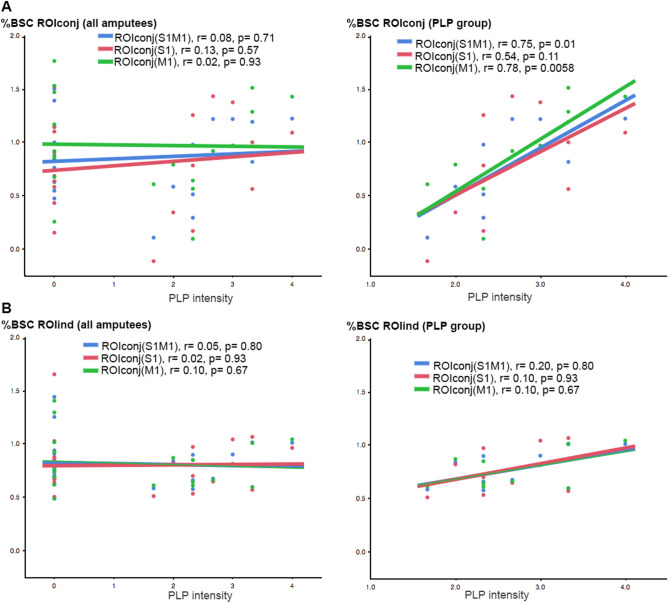
Correlation analyses between PLP intensity and %BSC in S1 (red), M1 (green) and S1M1 (blue) areas using **a** ROIconj and **b** ROIind in the entire sample of amputees (left) and in the PLP group only (right).

Using the entire sample of amputees and ROIconj, no significant relationship was found between PLP intensity and %BSC in S1 (r = 0.13, *p* = 0.57), M1 (r = 0.02, *p* = 0.93) or S1M1 (r = 0.08, *p* = 0.71), see Fig. 2a.

Examining the PLP group only, we found a significant positive relationship between PLP intensity and %BSC based on ROIconj in S1M1 (r = 0.75, *p* = 0.01, p_FDR_ = 0.03, 95% CI [0.23, 0.94]) as well as M1 (r = 0.78, *p* = 0.006, p_FDR_ = 0.03, 95% CI [0.33, 0.95]), but not for S1 (r = 0.54, *p* = 0.11, 95% CI [− 0.13, 0.87]), see Fig. 2b. We further computed bias-corrected and accelerated bootstrap 95% confidence intervals and associated p-values for the Pearson correlation coefficient for M1 [0.50, 0.95], *p* = 0.006, S1 [− 0.11, 0.87], *p* = 0.55, and S1M1 [0.43, 0.92], *p* = 0.14.

Using the entire sample of amputees and ROIind, no significant relationship was found between PLP intensity and %BSC in S1 (r = 0.02, *p* = 0.93), M1 (r = 0.10, *p* = 0.67), or S1M1 (r = 0.05, *p* = 0.80). Also in the PLP group no significant relationship was found between PLP intensity and %BSC in S1 (r = 0.10, *p* = 0.93), M1 (r = 0.10, *p* = 0.67), or S1M1 (r = 0.20, *p* = 0.80), see Fig. 2b.

We then examined if the correlation coefficients obtained for the ROIconj analysis were statistically different from ROIind. We found significant differences between the two correlation coefficients in S1M1 (two-tailed, Williams' test, t(7) = 3.69, *p* < 0.008) and M1 (t(7) = 4.97, *p *value < 0.002), but not for S1 (t (7) = 1.56, *p* = 0.16).

We then recalculated ROIconj based only on the controls with z > 40 (n = 12, Supplementary Fig. 3A) and carried out correlation analyses between %BSC in ROIconj (in M1, S1 and M1S1) and PLP intensity (Supplementary Fig. 3 B). Similarly to the correlations using the entire sample of controls, we found a significant correlation between %BSC and PLP intensity in the PLP group in the M1 ROI (r = 0.66, *p* = 0.037). We also reproduced the non-significant correlation between %BSC and PLP intensity in the S1 ROI (r = 0.34, *p* = 0.33). However, we did not reproduce the correlation between PLP intensity and %BSC in S1M1 ROI (r = 0.49, *p* = 0.15). In addition, similarly to the correlations using the entire sample of amputees, we did not find a relationship between %BSC and PLP intensity in M1, S1 or S1M1 ROIs (r < 0.14, *p* > 0.54). This analysis reproduced all the previous results in M1 and S1, but not in S1M1.

#### Correlation between EMG signal and fMRI activity

EMG was recorded in a subsample of 12 amputees (7 PLP, 5 nonPLP). There was no significant association between task-related neural activity in the hemisphere related to the virtual phantom movement and the EMG signal from the residual limb (n = 11, r = − 0.01, *p* = 0.95). One data set could not be retrieved.

#### Comparisons of perceptual data between groups

We did not find differences between the three groups in the perceptual ratings of the phantom/hand movement for the three items related to their sensations perceived during the task (KW test χ^2^ = 4.38, *p* = 0.11 for item1, χ^2^ = 5.73, *p* = 0.06 for item2 and χ^2^ = 2.14, *p* = 0.34 for item3). For item2 (vividness of movement), we found a significant difference between the controls and the PLP group, indicating that the PLP group had a more vivid perception of the phantom/hand movement compared with the controls (PLP:M ± SD = 3.86 ± 2.24; controls: 2.2 ± 1.50; KW test χ^2^ = 4.37, *p* = 0.036). However, the difference between the PLP and nonPLP groups (M ± SD = 2.30 ± 1.93) did not reach significance (KW test χ^2^ = 5.73, *p* = 0.057). We then tested if the increased activity that we found in amputees vs. controls in the hemisphere related to the virtual phantom/matched hand movement was related to increased vividness of perceived movement (item2): we carried out a whole-brain analysis comparing neural activity between controls and amputees (unpaired-t-test) using the ratings from item2 as a regressor. This analysis reproduced previous results, that is increased activity in S1 and M1 in amputees vs controls in the hemisphere representing the virtual phantom/matched hand.

#### Comparisons of cortical distances between groups

**Figure 3:**
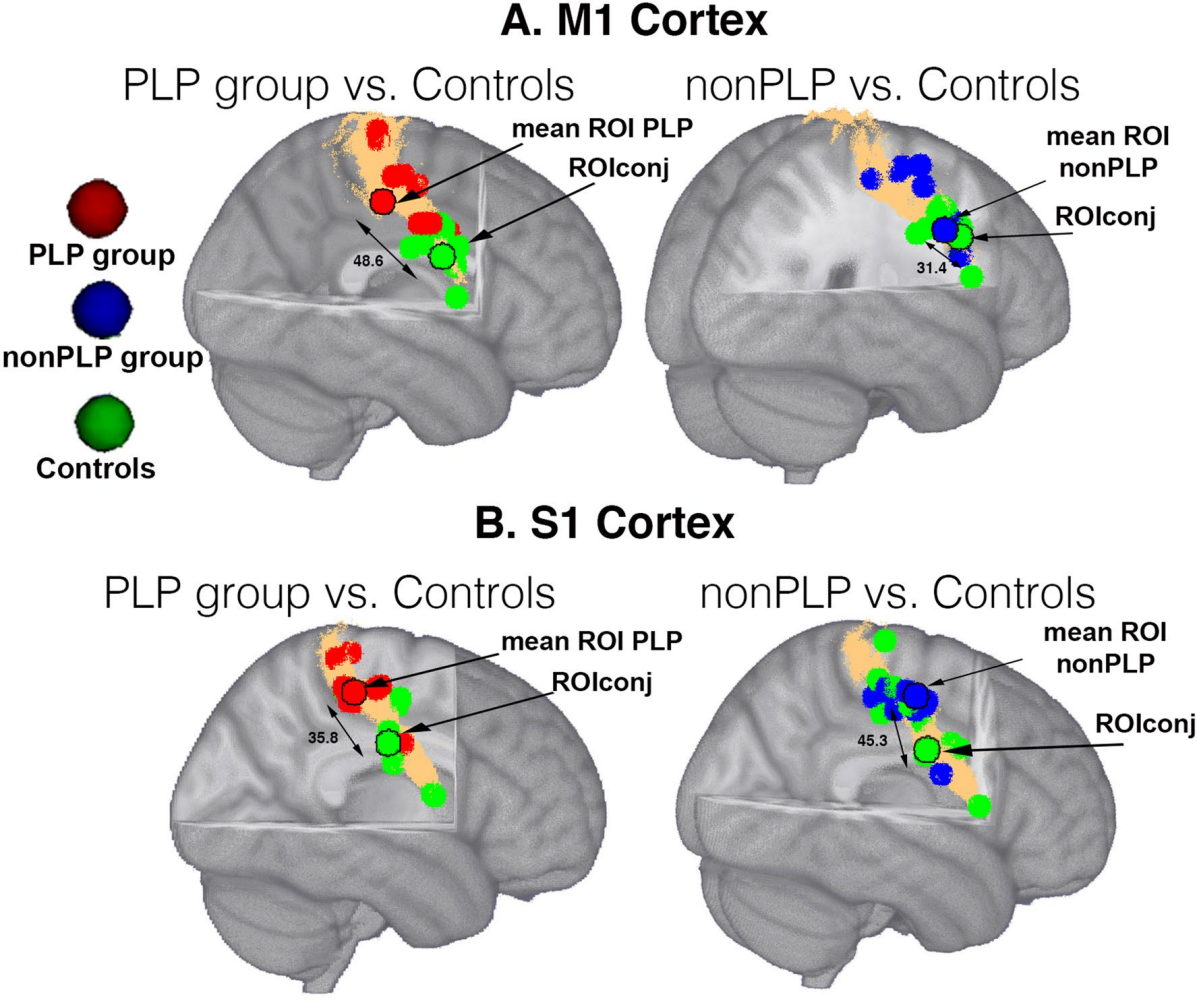
**a** Individual ROIs based on peak coordinates for the right hand ROI for the PLP (red), the nonPLP group (blue) and controls (green) in **a** the M1 cortex and **b** S1 cortex. The ROI for the controls overlapped entirely with ROIconj. The ROIs with a black contour indicate the mean of the individual ROIs. The surface coloured in copper indicates the mask of M1 (**a**) and S1 (**b**) defined by the Juelich atlas. Double-headed arrows indicate cortical distances in mm. ROIs are mapped on a MNI152 template provided by FSL.

Cortical distances between the ROIconj and ROIind differed between the PLP and the nonPLP groups in S1 (t(18) = 2.71, *p* = 0.01 p_FDR_ = 0.04, but not in M1 or S1M1 (t(18) < − 1.67, *p* > 0.11), see Fig. 3a,b). Data for the controls are not provided since the ROI for the controls completely overlapped with ROIconj.

For the entire sample of amputees, there was no significant relationship between PLP intensity and cortical distances between ROIconj and ROind for S1M1 (*r* = 0.22, *p* = 0.36), S1 (*r* = 0.27, *p* = 0.27), or M1 (*r* = 0.32, *p* = 0.18). Similarly, for the PLP group, no significant relationship between PLP intensity and cortical distances between ROIconj and ROind was found (*r* ≤ 0.31, *p* ≥ 0. 38).

There was also no significant relationship between PLP intensity and the cortical distances in the mediolateral axis between ROIconj and ROind in the entire sample of amputees for S1 (r = 0.40, *p* = 0.08), S1M1 (r = 0.28, *p* = 0.30) and it did not reach FDR correction for M1 (r = 0.45, *p* = 0.045, p_FDR_ = 0.12).

## Discussion

We showed that the neural representation of movements of the mirrored hand is different between amputees and non-amputated controls in terms of intensity and location of activity. These findings were only observed using ROIind and not using ROIconj.

### Task-neural activity between amputees and controls in ROIind and ROIconj

Using ROIind, amputees showed increased neural activity in S1, M1 and S1M1 in the hemisphere contralateral to the amputation compared with controls, which was also reported previously^20^. Using conjunction ROIs, there was no significant difference in task-related activity between amputees and controls, which is also comparable to previous findings^13^, where task-related activity of all amputees and controls was combined. In addition, there were no significant differences in task-related activity between amputees with and without PLP, using either ROIind or ROIconj. These both significant and non-significant results provide relevant information regarding the neural changes occurring after amputation. They suggest that although some neural activity remains in the “intact” hand area, the peak of activity in amputees is displaced dorsally and the degree of displacement depends on whether S1 or M1 was examined. The increased task-related activity in amputees versus non-amputated controls was obviously not specifically related to PLP since it was present in those with and without pain. Considering that only one hand was moving in the present study, the neural activity observed in S1M1 could be related to intact hand movements and not to phantom movements. However, when we compared task-related neural activity in the amputee group during the virtual reality movement task with the actual movement seen in the VR environment (no mirror), neural activity in the hemisphere contralateral to the amputation was not different compared to the mirror condition, while it disappeared in the hemisphere contralateral to the intact hand movements, demonstrating that the neural activity we observed is not related to intact hand movements (Supplementary Fig. S1). The higher activation in the mirror task could be related to stronger sensations of phantom movement and ownership. However, we found that only one of three items related to the percept of the phantom movement differed between amputees and controls, relating to vividness of movement. The PLP group reported a stronger vividness of perceived movement compared with controls. This difference could be related to a higher level of attention to the phantom limb^59^ or to the presence of phantom limb sensations^60^, which might lead to stronger immersion during the task.

### Relationship between task-related neural activity and PLP intensity

Using ROIconj, there was no significant relationship between PLP intensity and neural activity in M1, M1 or S1M1. However, when considering only amputees with PLP, we found a significant positive relationship between PLP intensity and neural activity in M1 and S1M1, but not in S1. Specifically, this relationship was only observed within the PLP, but not when the nonPLP group was included. Thus, activation in M1 and S1M1 seems to be positively related to the magnitude of PLP rather than the presence versus absence of PLP.

Figure 2 shows that there is no mean difference between amputees with and without pain in the ROIconj, but that those with low levels of PLP show lower and those with high levels of PLP show higher activation in the ROI derived from the conjunction of the three groups. This increased task-related activation might be indicative of higher excitability related to PLP. Alternatively, subjects with more severe PLP might have performed the task differently.

Our results emphasize the importance of differentiating between amputees with and without PLP. Only a minority of two of a total of 18 amputees was PLP-free in a previous study reporting a positive correlation between neural activity and PLP^13^ so that the correlation was potentially driven by the magnitude of PLP rather than its presence per se, similar to our results for the ROIconj. Furthermore, our results indicate that it is important to take the locations of neural activity, rather than its mere strength of activation, into account. In a sample of 27 amputees, including five without PLP, Kikkert et al.^15^ used a ROI defined by mean task-related activity in amputees. They replicated the positive correlation between neural activity in the missing hand area and PLP as previously reported ^13^. The ROI *location* for amputees with PLP was, however, not examined in this study, therefore potential reorganization related to PLP could not be assessed.

When we used individually defined ROIs, we could not find any relationship between PLP and percent signal change in S1, M1 or S1M1, neither when looking at all amputees, nor for the sub-group of amputees with PLP. This is in contrast to previous results^13^ showing that neural activity in the S1M1 contralateral to the missing limb was positively related to PLP intensity. We found that the consideration of individual variability in the location of S1M1 dispersed the correlation between S1M1 activity and PLP intensity, emphasizing the importance of considering inter-individual variability in the representation of the phantom limb.

Thus, we suggest that the definition of the ROIs as well as the composition of the amputee sample are important to assess the neural plastic changes occurring after limb amputation: While both conjoint versus individually-defined ROIs show divergent relationships with PLP intensity, they may also highlight different aspects of plastic changes.

### Role of the residual limb during the virtual phantom movement task

The neural activation we reported in the deafferented hemisphere was not due to associated activity in residual limb muscles since the task we used was implemented to specifically prevent residual limb muscle involvement. EMG recordings in a subgroup showed no significant relationship between muscle activity of the residual limb and brain activation in the virtual phantom movement task. Thus, we ensured that S1M1 activation was not confounded with muscle activity related to the residual limb. However, it has to be noted that only a subgroup of amputees underwent EMG recordings, so that this influence cannot be completely ruled out.

In this study we used a similar task for both amputees and controls, providing therefore homogenous conditions for assessing sensorimotor processes across amputees with and without PLP and controls. In a previous study^13^ the amputees were instructed to perform phantom movements, and if not possible, they used imagery both of which may actively involve the residual limb^26,29^. The neural activation reported in such studies might not only represent the phantom limb, but the area representing the residual limb adjacent to the missing limb representation^14,61^.

This hypothesis is supported by previous results^18^ suggesting that neural activity in the sensorimotor cortex does not only arise from residual muscles but also from other body parts such as the lips, elbow or feet. Electromyographic activity from the residual limb was also shown to be positively linked to PLP severity^62^. Such findings seem in opposition with the ones from Kikkert et al.^15^, who reported that residual limb activity was not associated with PLP, although these results were only reported for a ROI derived from both controls and amputees, lacking the definition of a specific ROI in amputees with PLP as in the present study.

In addition, an altered motor control could also “confound” neural activity in the sensorimotor cortex during phantom movements. For instance, the extent of the S1 hand representation was shown to be positively correlated with motor control over the phantom hand^19^. Amputees with PLP have been shown to have decreased phantom limb motor control^16,26^, which has been associated with increased task-related neural activity^51^ and cortical reorganization in the missing hand area^18^.

Furthermore, use-dependent effects cannot be ruled out. In this regard, residual limb and prosthesis use have been shown to be related to cortical structure, neural activity^28^ and functional connectivity^63^ and could be a potential driver of cortical reorganization. These use-dependent effects, however, did not interact with the relationship between PLP and increased neural activity in the missing hand area^15^. Therefore, factors such as residual limb activity and compensatory use might induce neural or plastic effects affecting the representation and/or activity of the phantom limb, and thus should be taken into account. In the presence of PLP, these factors might even play a bigger role.

### Cortical distances between maxima of activations

In the amputees with PLP, during perception of a moving phantom hand, the identified location of ROIind shifted depending on which cortical structure was being examined (i.e. S1 or M1). The cortical shift in the mediolateral direction was higher for S1 than for M1.

In controls, we found a rather stable cortical activation associated with the virtual contralateral hand movement task. The differences between motor and sensory cortices on the one side and between amputees and controls on the other side could be related to the different body map representations in primary somatosensory and motor cortices. Indeed, the body maps in M1 are far less fine-grained than the somatotopy found in S1^64–66^. It has been shown that body maps in M1 reflect movement types rather than individual body sites as in the S1 homunculus^64^. Moreover, we found that S1 somatotopy was more severely affected than M1 somatotopy in the PLP group as indicated by higher variance in the location of peak activity in S1. There was no significant difference in the somatotopy between S1 and M1 when amputees without PLP and controls were compared. Such differences in somatotopy could be possibly related to PLP interference of the presence of phantom limb sensations during the virtual phantom movement task^60,67^. This potential interplay, however, has to be examined in prospective studies.

### Role of execution versus imagery of phantom/mirror hand movements

The present findings suggest that although we used an identical virtual movement task for all subjects, the contribution of M1 and S1 varied between PLP and nonPLP, supporting differential roles of S1 and M1 as a function of presence or absence of PLP.

It is also important to underline that our virtual movement task is not an imagery task. The amputees were not asked to imagine moving their phantom hand. Rather, they were instructed to concentrate on the virtual representation of the moving phantom hand and to perceive it as their own phantom hand moving. Moreover, our findings are in line with previous literature implementing phantom movements execution^18,20,21,26^. Motor imagery and motor execution have been shown to rely differentially on primary sensory and motor processing, with the primary sensorimotor cortex being more involved during motor execution and parietal and occipital lobes being more involved during motor imagery^26,68^. Such differences are accentuated by the fact that execution of phantom movements is slower compared to imagery of phantom movements, possibly due to a lack of expected sensory feedback, while there is no feedback expectation during imagination^67^. In addition, PLP is often triggered or intensified by motor execution, which is not the case for motor imagery^67^, which might lead to different patterns of neural activity between amputees with and without PLP related to the neural activation of the phantom percept. However, we were not able to examine such effects, since we did not assess task-dependent PLP in the present study. Raffin et al.^26^ showed that phantom movements can be clearly distinguished from imagined phantom movements by demonstrating that amputation leads to a significant deceleration of movement speed as measured behaviorally and psychometrically. Moreover, the presence of phantom limb sensations seems also to necessitate more efforts and to lead to longer reaction times when performing a mental rotation task^60^. Further, Raffin et al.^26^ reported no significant electromyographic activity in residual limb muscles during motor imagery, indicating no incongruent afferent feedback of motor intentions from the residual limb.

Noteworthy, differences in neural activity during execution and imagery of phantom hand movements could be related to the presence of PLP. For instance, movements of the elbow and lips showed a shift of neural activity in the former hand representation area, which was related to PLP intensity^18^. Similarly, movements of the lips in PLP showed a shift of the lip representation in the deafferented primary and somatosensory cortices, which was also related to PLP intensity^20^. Using imagery of phantom movements, an increased task-related activity was shown in the hand and in the face cortical area in PLP and not in non-PLP, suggesting co-activation of the mouth and hand representations in primary M1 and S1 in PLP^20^. In addition, using mirrored movements of the intact hand to mimic phantom hand movements, an increased task-related neural activity was reported in M1 and S1 cortices representing the phantom hand in nonPLP but not in PLP^22^.

These findings indicate differences in cortical representation of the phantom hand depending on the task, i.e., imagery or execution of phantom movements, and also between amputees with and without PLP, therefore arguing for not using a common ROI. In addition, the studies reviewed above assessed neural activity in both motor and somatosensory cortices, but they did not examine potential differences between the contribution of motor versus sensory processes, which might make sense regarding neural processes involved in phantom movements and/or PLP. Differences in cortical representation might also be influenced by whether one examines somatosensory activity related to a sensory stimulation (e.g. tactile) or to motor movement, which follows a different somatotopy.

### Methodological considerations

We measured cortical distances in the mediolateral axis between activation peaks across a folded cortical volume, which is a standard method to assess cortical reorganization^10,69,70^. There is, however, increasing interest to consider individual cortical folding patterns since some studies showed that geodesic distances offer more accurate measurements along the cortex than cortical distances based on volume-based analyses^71,72^. The two measures have, however, been shown to be significantly correlated in the sensorimotor cortex^17^, as well as in S1 and M1, which have been examined separately before^71^. Moreover, no significant difference in the distance changes between the local maxima of M1 and S1 were found using volume- or surface-based analysis^71^.

Noteworthy, measures of cortical surface also suffer from limitations that restrict their interpretability. Measures of surface areas, for instance, do not provide information on the tissue or cell type being affected. Recent advances in multimodal imaging should enable a more precise characterization of the nature of such structural alterations^73^.

In addition, the size of the ROI might also affect the results we reported here. We used ROIs of 5 mm radius, which is comparable to previous studies with similar voxel size^74,75^. Differences between methods used to define the ROIs (e.g. based on anatomical structure, neural activity, group means, or on individual peak maxima measures) might induce more variability than the ROI size itself^76^.

Moreover, we restricted the S1 map to BA3b based on the neural activity induced by the virtual phantom/hand movement. We defined BA3b using the Juelich histological atlas (derived from human post-mortem brains allowing for the creation of probabilistic ROIs) since it provides subdivisions of S1 (BA1, BA2, BA3a, BA3b) whereas other atlases do not (e.g. AAL Automated Anatomical Labeling^32^, or the Harvard–Oxford^77^). Although the choice of an atlas might not be a concern within a single study since the same brain area is examined across multiple subjects, it should be noted that discordant parcellation methods might pose potential challenges, particularly for meta-analyses. A quantitative understanding of the correspondence between different parcellations methods might provide the necessary information for best-reconciling heterogenous reported results^34^.

We defined S1 and M1 based on the Juelich histological atlas in order to dissociate S1 and M1 representations. Although our ROIs are based on probabilistic maps, the distances (mean ± SD) between the S1 and M1 ROI peak maxima are on average 25.70 ± 17.37 mm (cortical distances in the antero-posterior axis), indicating different neural activity patterns across the ROIs. These findings should, however, be interpreted with caution. The ability to reliably dissociate S1 and M1 contributions to the resulting clusters is limited by the low spatial resolution of fMRI at 3 T and inaccuracies induced by data preprocessing and analyses. For instance, strong neural activations for hand motor tasks have been shown to be distributed near the adjacent regions (omega knobs) of M1 and S1^78^, leading to partial volume artefact and erroneous signal intensity. Moreover, the activation maps are derived from smoothed functional images, a procedure known to increase the sensitivity of detection of activation^79,80^ but it may also lead to probabilistic local maxima of activations that are artificially shifted^81,82^.

Moreover, we used a standard procedure consisting in “matching” the hemisphere contralateral to amputation across amputees^15,22,83^. To ensure that this procedure did not affect our results, we compared task-related neural activity and cortical distances between left and right amputees. There were no significant differences in task-related neural activity between left and right amputees in the hemisphere contralateral to amputation, using either an S1M1, S1 or M1 ROI (F(1,18) < 3.9, *p* > 0.06). There were also no significant differences in cortical distances between left and right amputees in the hemisphere contralateral to amputation, using either an S1M1, S1 or M1 ROI (F(1,18) < 0.88, *p* > 0.37). Therefore, the flipping procedure carried out to “match” the hemisphere contralateral to amputation across amputee should not have affected the results.

## Conclusion

We found that motor and sensory cortical areas show different activation patterns for amputees and healthy controls dependent on the definition of the region of interest. When individual ROIs were used, we found a positive relationship between the medio-to-lateral location in M1 and PLP intensity. Moreover, the amputees showed higher activation compared to controls in M1 and S1M1 when conjunction ROIs were used. M1 and S1M1 activation showed a relationship with PLP severity only in the PLP group and only when conjunction ROIs were used. Thus, we suggest that the term “sensorimotor” should be used with caution, especially when related to amputation and alterations in and M1 should be considered. We could show that deafferented motor and somatosensory body maps are differently involved in a sensorimotor task. Moreover, we also accounted for subject and group-wise variability in body site representation by systematically using ROIs based on the peak of neural activity and comparing measures derived from motor and somatosensory site cortical representation.

Finally, we focused on alterations in motor and somatosensory pathways following amputation, but we cannot exclude that these alterations might extend to other primary or associative areas such as visual cortex, or temporo-parietal cortex^84^. Longitudinal studies are needed to examine how functional reorganization differs between S1 and M1 in terms of extent, peak activation and speed, and its relationship with PLP. A better understanding of the role of M1 or S1 in PLP could help to optimize the definition of neural sites to be targeted using techniques such as transcranial magnetic stimulation or neurofeedback.

## Supplementary information

Supplementary information
