# Supplementary material for: Assessment of cortical reorganization and preserved function in phantom limb pain: a methodological perspective
Source: Sci Rep. 2020 Jul 13;10:11504. doi: 10.1038/s41598-020-68206-9 (PMC7359300; doi:10.1038/s41598-020-68206-9)
Supplement: Supplementary file 1 — Supplementary information [file 41598_2020_68206_MOESM1_ESM.docx]

**Supplementary material**

J. Andoh, C. Milde, M. Diers, R. Bekrater-Bodmann, J. Trojan, X. Fuchs, S. Becker, S. Desch, H. Flor. “Assessment of cortical reorganization and preserved function in phantom limb pain: a methodological perspective”

***Figure Supp.1.*** *Task-related neural activity in the hemisphere contralateral to intact hand activity (L) in (A) controls (green), in (B) nonPLP (blue) and in (C) PLP (red) groups.*


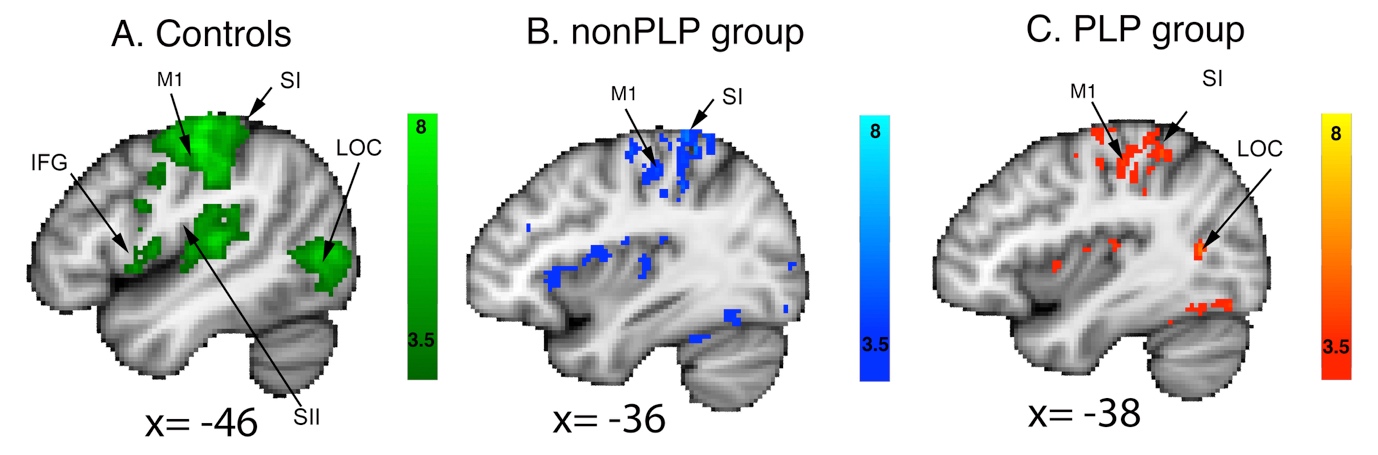


***Figure Supp.2.*** *Analysis (paired-t-test) removing the contribution of intact hand movements in the amputee group (contrast between virtual mirror hand movements and virtual intact hand movements, FWE p<0.05, TFCE). Neural activity did not change in BA4p and BA3b in the hemisphere ipsilateral to intact hand movements (R) and disappeared in the hemisphere contralateral to intact hand movements (L). The green circle indicates the location of the primary motor cortex.*

*
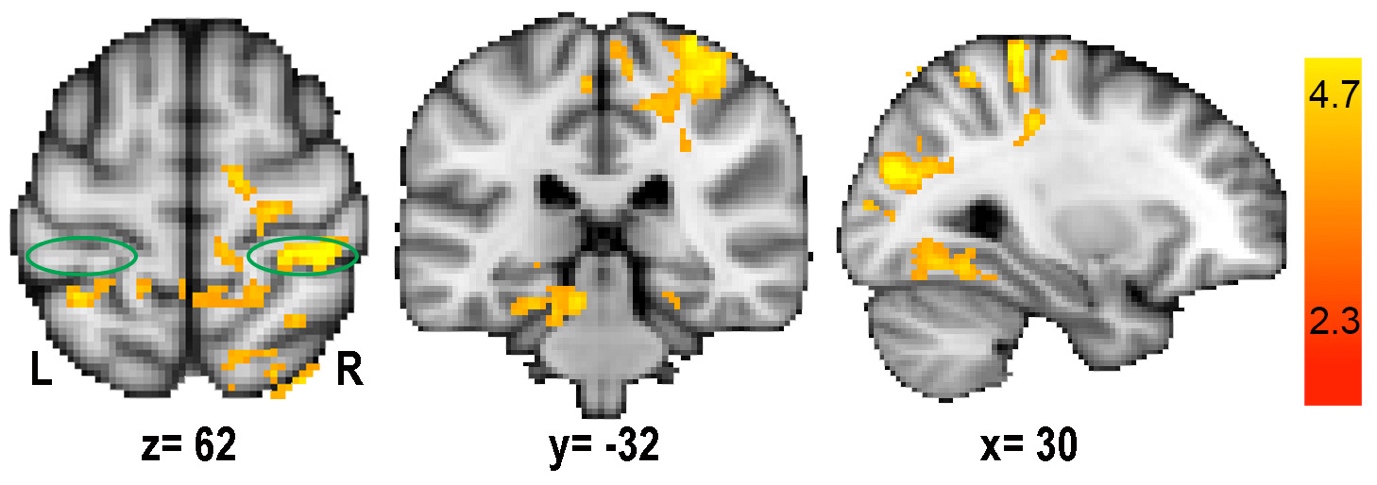
*

***Figure Supp.3.*** *(A) Individual ROIs for amputees with PLP (red) and controls (green) based on ROIconj defined from a subsample of the controls (n=12, with z>40). (B) Correlation analyses between PLP intensity in the PLP group and %BSC in M1 (green), in S1 (red), and S1M1 (blue) areas.*

***
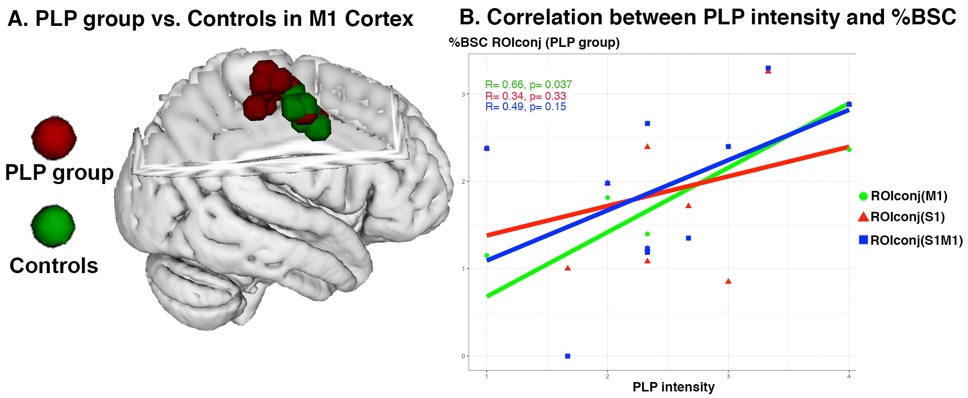
***

***Table Supp.1.*** *Mean % Bold Signal change (%BSC) and standard deviation (SD) for ROIconj and ROIind across groups. Abbreviations: AMP: amputees (PLP and nonPLP), PLP group: amputees with PLP, nonPLP group: amputees without PLP.*

| **ROIs** | **Groups** | **BSC (%) mean ± SD** | | |
| --- | --- | --- | --- | --- |
|  |  | **S1** | **M1** | **S1M1** |
| ROIconj | AMP | 0.79 ± 0.11 | 0.97 ± 0.12 | 0.85 ± 0.10 |
|  | Controls | 0.57 ± 0.11 | 0.68 ± 0.12 | 0.61 ± 0.10 |
|  | PLP group | 0.79 ± 0.15 | 0.88 ± 0.14 | 0.81 ± 0.12 |
|  | nonPLP group | 0.80 ± 0.15 | 1.07 ± 0.14 | 0.89 ± 0.12 |
| ROIind | AMP | 0.80 ± 0.05 | 0.82 ± 0.05 | 0.81 ± 0.05 |
|  | Controls | 0.50 ± 0.05 | 0.58 ± 0.05 | 0.55 ± 0.05 |
|  | PLP group | 0.78 ± 0.09 | 0.77 ± 0.08 | 0.77 ± 0.08 |
|  | nonPLP group | 0.82 ± 0.09 | 0.86 ± 0.08 | 0.85 ± 0.08 |

***Table Supp.2****. Mean Euclidean distances (D) and standard deviation (SD) between ROIconj and ROIind across groups. Abbreviations: AMP: amputees (PLP and nonPLP), PLP group: amputees with PLP, nonPLP group: amputees without PLP.*

| **Groups** | **D (mm) mean ± SD** | | |
| --- | --- | --- | --- |
|  | **S1** | **M1** | **S1M1** |
| AMP | 40.29 ± 3.30 | 38.77 ± 3.24 | 43.79 ± 4.87 |
| Controls | 19.74 ± 3.40 | 7.06 ± 4.94 | 25.88 ± 5.21 |
| PLP group | 35.83 ± 3.07 | 48.60 ± 4.17 | 47.35 ± 3.88 |
| nonPLP group | 45.32 ± 3.2 | 31.39 ± 3.61 | 40.62 ± 4.11 |
